# Natural compounds proposed for the management of non-alcoholic fatty liver disease

**DOI:** 10.1007/s13659-024-00445-z

**Published:** 2024-04-01

**Authors:** Théodora Merenda, Florian Juszczak, Elisabeth Ferier, Pierre Duez, Stéphanie Patris, Anne-Émilie Declèves, Amandine Nachtergael

**Affiliations:** 1 Unit of Clinical Pharmacy, Research Institute for Health Sciences and Technology, University of Mons (UMONS), Mons, Belgium; 2 Department of Metabolic and Molecular Biochemistry, Research Institute for Health Sciences and Technology, University of Mons (UMONS), Mons, Belgium; 3 Unit of Therapeutic Chemistry and Pharmacognosy, Research Institute for Health Sciences and Technology, University of Mons (UMONS), Mons, Belgium

**Keywords:** Non-alcoholic fatty liver disease, Natural compound, Sylimarin, Curcumin, Biochanin A, Oleanolic acid, Ginsenoside K, 6-Gingerol, Naringenin, Hesperidin, *Chenopodium quinoa*

## Abstract

Although non-alcoholic fatty liver disease (NAFLD) presents as an intricate condition characterized by a growing prevalence, the often-recommended lifestyle interventions mostly lack high-level evidence of efficacy and there are currently no effective drugs proposed for this indication. The present review delves into NAFLD pathology, its diverse underlying physiopathological mechanisms and the available in vitro*, *in vivo, and clinical evidence regarding the use of natural compounds for its management, through three pivotal targets (oxidative stress, cellular inflammation, and insulin resistance). The promising perspectives that natural compounds offer for NAFLD management underscore the need for additional clinical and lifestyle intervention trials. Encouraging further research will contribute to establishing more robust evidence and practical recommendations tailored to patients with varying NAFLD grades.

**Graphical Abstract:**

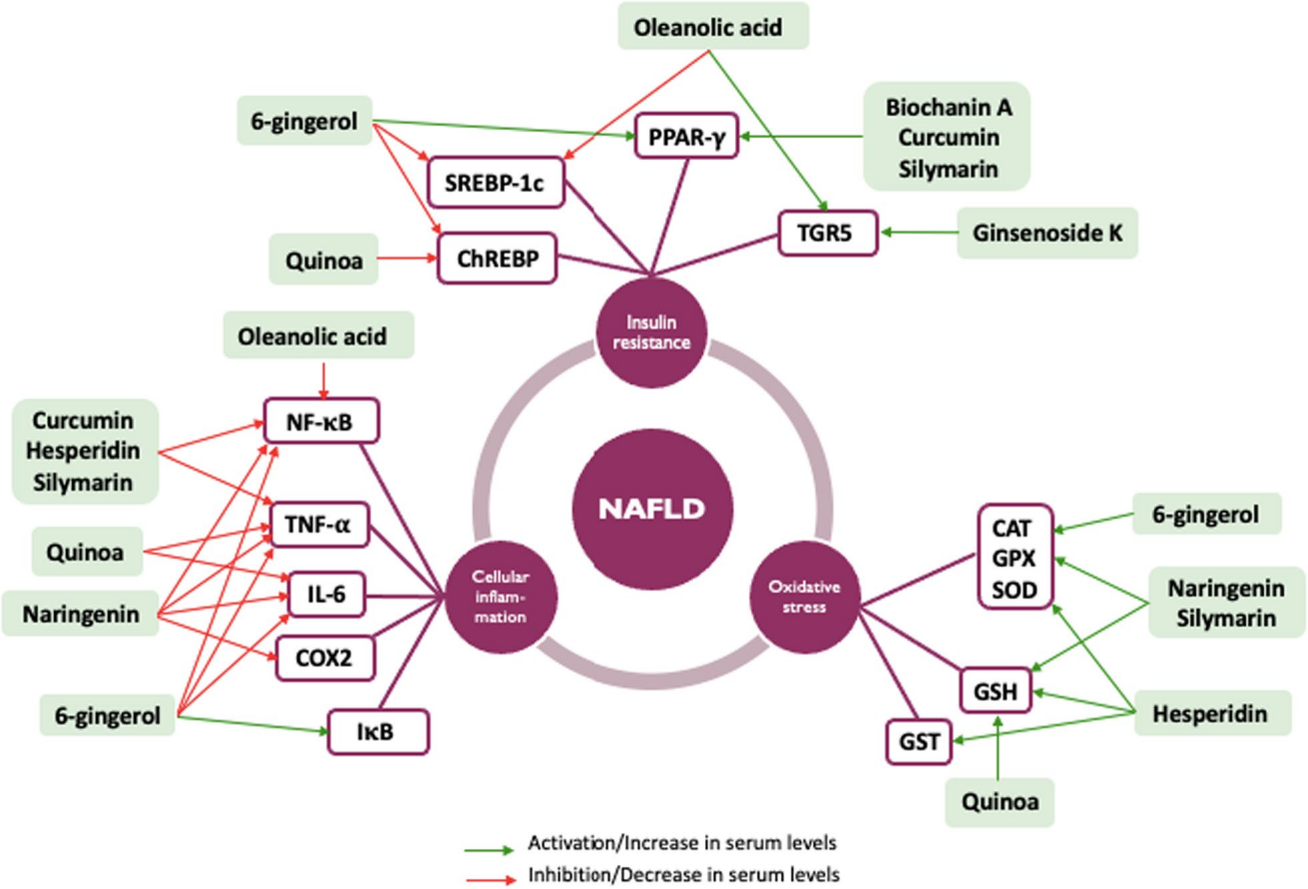

## Introduction

Non-alcoholic fatty liver disease (NAFLD), the most common liver disease in industrialized countries, is expected to expand with the continuous increase in obesity epidemics [1–3]; moreover, NAFLD has been associated with an increased risk of type 2 diabetes (T2DM) and cardiovascular disease (CVD). Most patients with NAFLD usually exhibit pathological traits such as obesity, insulin resistance, hypertension, and hyperlipidemia. Therefore, NAFLD is also referred to as “*metabolic dysfunction-associated fatty liver disease*” (MAFLD) [4]. Long-term NAFLD may initiate the development of non-alcoholic steatohepatitis (NASH), which will likely progress to cirrhosis and hepatocellular carcinoma (HCC) [5–7]. NAFLD is thus becoming a serious health and economic burden as well as a major concern for hepatologists since there is currently no effective drug with a marketing authorization for this indication [1, 6, 8]. Although NAFLD has attracted the attention of pharmaceutical companies for the development of new drugs, its management is currently only based on the implementation of an appropriate diet, regular physical activity, and etiological treatment such as e.g. bariatric surgery [2, 6, 9]. In an interesting approach, several research teams have been investigating the efficacy of natural compounds for the treatment of NAFLD. By focusing on critical elements within the disease pathophysiology, these compounds could provide a novel opportunity to positively influence disease progression, potentially halting the advancement of NAFLD.

## Histological spectrum and diagnosis of NAFLD

**Fig. 1:**
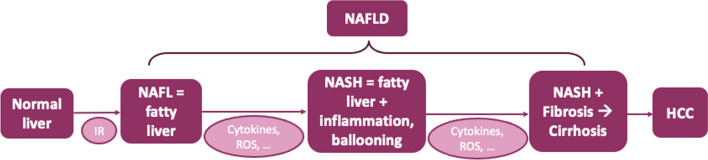
Histological spectrum of NAFLD. *H0CC* hepatocellular carcinoma, *IR* insulin resistance, *NAFL* non-alcoholic fatty liver, *NAFLD* non-alcoholic fatty liver disease, *NASH* non-alcoholic steatohepatitis, *ROS* reactive oxygen species

Primary NAFLD is characterized by an abnormal and excessive accumulation of triglycerides (TGs) within hepatocytes [5, 6, 10] that is not related to alcohol abuse or steatosis factors such as viral infections or hepatotoxic drugs [5, 10]. The histological features of the disease (Fig. 1) range from simple steatosis (NAFL) to non-alcoholic steatohepatitis (NASH). NAFL corresponds to fatty liver, characterized by lipid droplets in more than 5% of hepatocytes, but without lesions. NASH, the advanced and severe manifestation of NAFLD, is characterized by the presence of steatosis accompanied by hepatocyte ballooning. This hepatocyte ballooning involves an enlargement of hepatocytes and their clarification. Additionally, NASH includes lobular inflammation, which consists of the infiltration of lymphocytes, macrophages, and neutrophils, as well as focal hepatocyte necrosis [11, 12]. NASH may evolve into liver cirrhosis and/or progress to end-stage liver disease and hepatocellular carcinoma [10].

The gold standard method for NAFLD diagnosis is liver biopsy [6]. However, this technique cannot be performed in all patients because of its invasive and painful nature, some rare complications, and the high prevalence of the disease [13, 14]. Noninvasive methods based on serum markers and imaging have therefore been developed. Table 1 briefly summarizes the different tests that can be used for diagnosis. Henceforth, MAFLD is considered by a consortium of international liver specialists as a standalone disease, which implies setting new diagnosis criteria [4], i.e. (i) a liver steatosis confirmed by one of the tests mentioned in Table 1 and (ii) overweight or obesity (BMI ≥ 25 kg/m^2^ in Caucasians and 23 kg/m^2^ in Asians), or T2DM [4]. These metabolic conditions accompanying liver steatosis are sufficient to diagnose NAFLD [4]. Under other metabolic conditions, a NAFL accompanied by at least two metabolic abnormalities, which are defined in Table 2, are needed to set a NAFLD diagnosis [4].

**Table 1:** The different diagnostic methods of the NAFLD [6, 13, 14, 30, 137]

Patients with steatosis	FLI: detection and confirmation of steatosis, identification of people at higher risk of developing NAFLD (sensitivity = 83%; specificity* = *70*%*) ^1^H-MRS: reference method but high cost and time consuming Ultrasound-based radiography (use a sound wave): recommended first-line method for a rapid appreciation of the liver structure and lesions
Patients at low risk of advanced fibrosis and cirrhosis	NFS and FIB-4 index (simple blood tests): recommended as first-line to discriminate advanced fibrosis stage F3/F4 (specificity = low) Fibrotest, fibrometer, hepascore and ELF test (specialized blood tests that gives an indication on the fibrosis severity): better diagnostic value (sensitivity = high) Fibroscan (pulse elastography, use a low amplitude wave): measurement of liver “hardening” (liver stiffness, inflammation and, fat) MRE (use of an MRI scan): identify whole organ fibrosis → The combination of a blood test and fibroscan increases the accuracy of diagnosis and thus limits the need of liver biopsy confirmation
Patients with NASH	Liver biopsy (histological diagnosis): gold standard method for identifying fibrosis and inflammation

*ELF test* enhanced liver fibrosis test, *FIB-4 index* fibrosis-4 index, *FLI* fatty liver index, ^*1*^*H-MRS* magnetic resonance proton spectroscopy, *NFS* NAFLD fibrosis score, *MRE* magnetic resonance elastography, *MRI* magnetic resonance imaging

**Table 2:** Summary of the metabolic abnormalities that may accompany hepatic steatosis in adults considered to set a diagnosis of NAFLD [4]

Waist circumference	**≥ **102/88 cm in Caucasians (or ≥ 90/80 cm in Asians)
Blood pressure	**≥ **130/85 mmHg or specific drug treatment
Plasma triglycerides	**≥ **150 mg/dL (≥ 1.70 mmol/L) or specific drug treatment
Plasma HDL-cholesterol	**< **40 mg/dL (< 1.0 mmol/L) for men and < 50 mg/dL (< 1.3 mmol/L) for women or specific drug treatment
Prediabetes	i.e., fasting glucose levels 100 to 125 mg/dL [5.6 to 6.9 mmol/L], or 2-h postload glucose levels 140 to 199 mg/dL [7.8 to 11.0 mmol] or HbA1c 5.7% to 6.4% [39 to 47 mmol/mol]
Homeostasis model assessment of IR score	**≥ **2.5
Plasma high-sensitivity C-reactive protein level	**> **2 mg/mL

*HDL* high-density lipoprotein, *HbA1c* hemoglobin A1c, *IR* insulin resistance

## Incidence, prevalence, and risk factors for NAFLD

The incidence and prevalence of NAFLD, although difficult to accurately estimate due to variations in diagnosis methods [5, 12], have significantly increased since the twentieth century and are expected to rise over the coming years both in Western and in developing countries. Although there is a lack of data regarding the incidence of NAFLD [3, 7, 15], the prevalence of the disease, estimated from ultrasound imaging diagnosis, is variable across continents, countries and regions: in Europe, the average prevalence is 25.1% (range, 20.55 to 30.28%, depending on the country); in the USA, the prevalence is estimated at 31% with variations according to ethnicity, as it is higher in Hispanic Americans and lower in European and African Americans; in Latin America, the prevalence of NAFLD is estimated at 44.4% with variations according to countries. The average prevalence in other parts of the world is approximately 36.5% in the Middle East, 31% in Asia, and approximately 20% in Africa [3, 16, 17].

Several factors increase the risk of developing NAFLD; the prevalence of the disease is notably higher in obese patients (> 95%), diabetic patients (33–66%), patients with dyslipidemia (50%) and patients with metabolic syndrome (MetS) and polycystic ovary syndrome. Lifestyle is an important parameter to consider as an increase in sedentary lifestyle, and a high consumption of sugars and saturated fatty acids is correlated with an increase in NAFLD. Moreover, the prevalence of NAFLD increases in people over 50 years old [3, 6, 10, 18].

## Pathogenesis of NAFLD

**Fig. 2:**
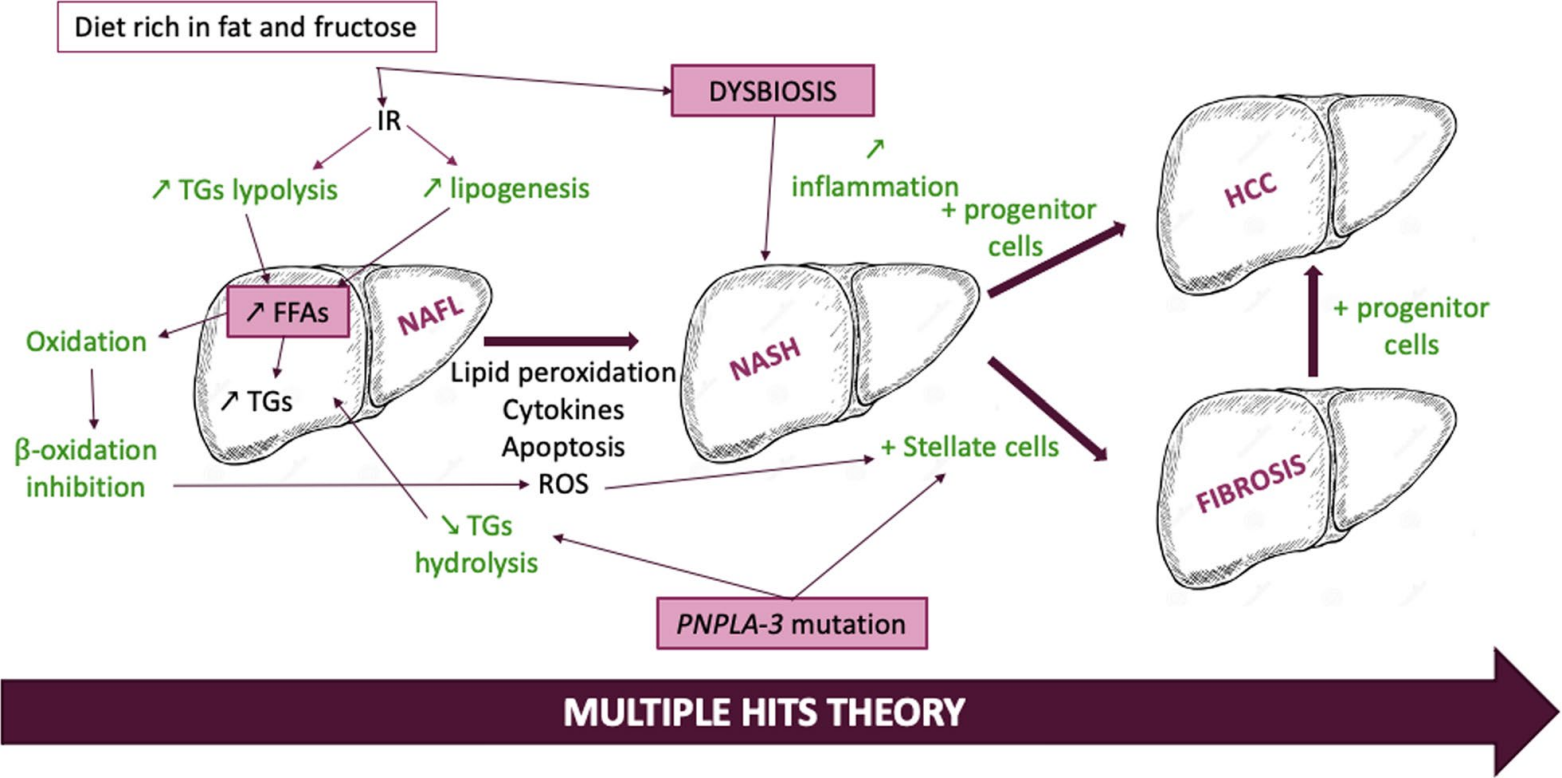
Pathogenesis of NAFLD. *FFAs* free fatty acids, *HCC* hepatocellular carcinoma, *IR* insulin resistance, *NAFL* non-alcoholic fatty liver, *NASH* non-alcoholic steatohepatitis, *PNPLA-3* patatin-like phospholipase domain-containing protein 3, *ROS* reactive oxygen species, *TGs* triglycerides

The pathogenesis of NAFLD, complex and still poorly understood, is thought to be the result of “*multiple hits*” along different metabolic pathways (Fig. 2) [2, 19].

### Nutritional factors

The chronic elevation of circulating lipids and/or carbohydrates in obesity or type 2 diabetes leads to an increase in fat storage in nonadipose tissues, especially in the liver, an organ particularly affected by excess caloric intake because of its central role in lipid and glucose metabolism. This phenomenon is known as “*ectopic fat accumulation*” [20]. Under these conditions, the hepatocyte plasma membrane exhibits an increase in fatty acid transport proteins, such as FA-transport and FA-binding proteins, and the activation of two transcription factors, sterol regulatory element binding protein (SREBP-1c) and carbohydrate responsive element binding protein (ChREBP), which activate local de novo lipogenesis [21, 22]. This increased hepatic lipogenesis leads to the accumulation of free fatty acids (FFAs), which are stored as TGs and exported as very low-density lipoprotein particles (VLDLs). At the onset of the disease, the FFAs from lipid droplets enter mitochondrial β-oxidation to provide cellular energy. However, during the progression of the disease, fatty acid deposition exceeds the liver capacity to metabolize and export FFAs. Additionally, an inhibition of β-oxidation is associated with decreased expression of peroxisome proliferator-activated receptor-α (PPAR-α), a transcription factor crucial for the expression of proteins involved in FFA transport and β-oxidation [23]. Furthermore, the accumulation of FFAs alters the normal function of mitochondria, lysosomes, and endoplasmic reticulum, notably through the activation of intracellular pathways that promote mitochondrial and lysosomal membrane permeabilization, oxidative stress, and inflammation, which can lead to hepatocyte apoptosis [24]. The combination of these 4 elements (increased lipid influx and de novo lipogenesis; decreased β-oxidation and lipid efflux) leads to steatosis, induces cell damage, and promotes cellular necrosis, inflammation, fibrosis, and cirrhosis [12, 19, 20].

### Gut microbiota

The gut microbiota plays an important role in the development of NAFLD. Indeed, in people with NAFLD, the intestinal microbiota becomes less diverse and gram-negative bacteria become more abundant [25]. Bacteria are involved in the metabolization and enterohepatic cycling of bile acids, important digestive components that notably allow the solubilization and absorption of dietary lipids and fat-soluble vitamins [26]. But, as a low level of plasmatic bile acids disrupts the activity of different receptors (G protein-coupled receptor 5, TGR5, and Farnesoid X receptor, FXR), alterations of bile acid metabolism and composition have been shown to promote the development of NAFLD [26, 27]. Obesity and diabetes have been associated with dysbiosis, which might contribute to the development and progression of NAFLD [28, 29]. Dysbiosis induces the release of proinflammatory cytokines such as interleukins (IL-6 and IL-12) and TNF-α by macrophages and thus generates an intrahepatic inflammatory response contributing to the progression of NAFLD [19, 30]. Also, dysbiosis alters another metabolic function of bacteria from the intestinal flora, namely, their ability to produce short-chain fatty acids (acetate, propionate, and butyrate) from dietary fibers, leading to increased blood sugar, IR, and inflammation but also to a decrease in the production of glucagon-like peptide 1 (GLP-1) [26, 30].

### Genetic factors

The hereditary component of NAFLD is estimated at between 35 and 61% [25]. A robust relationship between genetic factors and the development of NAFLD has been established in a genome-wide association study (GWAS) [31]. One of the most important identified genes is *PNPLA-3* and its *PNPLA-3* I148M variant [32, 33]. The *PNPLA-3* gene encodes a patatin-like protein, highly expressed in the liver and retina, with TGs and retinyl ester esterase activity. The expression of this gene seems to be regulated by nutritional factors [34, 35]. The lipid esterase activity (lipase) of the I148M variant is altered, which would induce a decrease in TG hydrolysis and thus a sequestration of fats in hepatocytes and stellate cells with an increase in droplet size favorable to the development of the disease [33, 36, 37]. This mutated enzyme accumulates in lipid droplets and is frequently found in Hispanic and less so in African populations [31, 32, 38, 39]. It should also be noted that this variant is not associated with changes in insulin sensitivity [38]. Other genes are involved in the development of NAFLD. The polymorphism *rs641738* reduces expression of membrane-bound *O*-acetyltransferase domain containing 7-trans-membrane channel-like 4 (*MBOAT7-TMC4*) and is associated with increased liver lipid content [25]. Also, several polymorphisms in ApoC3 can lead to hypertriglyceridemia, insulin resistance and NAFLD [25].

### Complications

As mentioned before, NAFLD is a slowly progressing multisystem disease, which means that it can lead to many hepatic and extrahepatic complications [2, 5]. Hepatic complications include a progression of the disease to NASH in a certain number of patients, depending on environment and genetics [2, 12] and HCC. The likelihood of developing HCC is higher in patients affected by NASH with cirrhosis than in patients with NAFLD [6]; an increased prevalence of HCC may be related to genetics, including the I148M variant of the PNPLA-3 gene, which represents a significant cause of mortality in NAFLD patients. Regarding extrahepatic complications, NAFLD often leads to cardiovascular diseases, explaining the necessity of NAFLD diagnosis in any patient with a cardiovascular history [6]. Indeed, a disturbance of lipoprotein metabolism in hepatocytes can lead to the ectopic accumulation of fat in the myocardium, which would lead to the deregulation of heart function. Similarly, via ectopic accumulation of fat in the kidneys, chronic kidney diseases are also a major complication of NAFLD. It should also be noted that extrahepatic cancers are among the top three causes of death in patients with NAFLD [30, 40, 41]. Furthermore, this disease is associated with a higher risk of developing T2DM, which is why it is mandatory to screen a patient with the disease for diabetes. Conversely, type 2 diabetic patients may more easily develop NAFLD in association with the presence of IR [6].

## Current treatment options

The management of NAFLD is based on three pillars that aim at slowing down the development of the disease and its progression to NASH: (i) lifestyle intervention, (ii) pharmacological therapies and (iii) surgical intervention.

The first-line intervention for the clinical management of NAFLD is based on lifestyle changes. First, the adoption of an appropriate diet to achieve weight loss and thus reduce hepatic fat accumulation with energy restrictions (avoidance of high-fat meals and elimination of food rich in fructose) [2, 6]. Second, the practice of regular physical activity decreases inflammation and increases the level of irisin (myokine), a hormone that mediates weight loss and thermoregulation [42]. It is worth noting that combining diet modification and the increase in physical activity would be more effective [2, 6, 12, 43].

The therapies used in the management of NAFLD aim to improve steatosis and prevent disease evolution to NASH but do not have a marketing authorization for this indication [2]. Thiazolidinediones (pioglitazone and rosiglitazone) improve hepatic insulin sensitivity and decrease hepatic TG content through activation of PPAR-γ [44]. They significantly decrease steatosis, hepatocellular ballooning, and inflammatory activity in NASH [8, 45]. Incretin mimetics (GLP-1 analogues such as liraglutide) act on the glucose-insulin interaction and increase meal-related insulin secretion [46]. Statins induce a reduction in hepatic steatosis and inhibit the progression of steatosis to NASH, but these drugs have not been sufficiently evaluated for this indication [47, 48]. Obeticholic acid improves both IR and NASH in terms of inflammation, ballooning, and fibrosis [8, 49]. Vitamin E has shown improvement in steatosis, inflammation and ballooning as well as regression of NASH in nondiabetic [45, 50] and noncirrhotic patients [51]. There is no extensive evaluation of the use of vitamin E in diabetic patients. Polyunsaturated fatty acids (including omega 3) may induce a decrease in liver steatosis and injury, as in liver and blood lipid levels, but evidence is conflicting as to their beneficial effects [52–56].

Stearoyl-CoA desaturase-1 (SCD-1) modulators, such as icomidocholic acid, both decrease lipogenesis and hypertriglyceridemia and enhance β-oxidation of fatty acids and insulin sensitivity in NAFLD patients [57].

Bariatric surgery can be used in patients for whom lifestyle modifications and drug treatments are ineffective but also in patients with BMI ≥ 40 kg/m^2^ (morbid obesity) or ≥ 35 kg/m^2^ with at least one comorbidity. A systematic literature review indicated a reduction in necrosis and inflammation and an improvement in fibrosis following bariatric surgery [58].

Finally, liver transplantation is the main treatment option for patients with end-stage NASH as NAFLD with cirrhosis is among the main conditions requiring liver transplantation [6].

## Future direction for the management of the disease

**Fig. 3:**
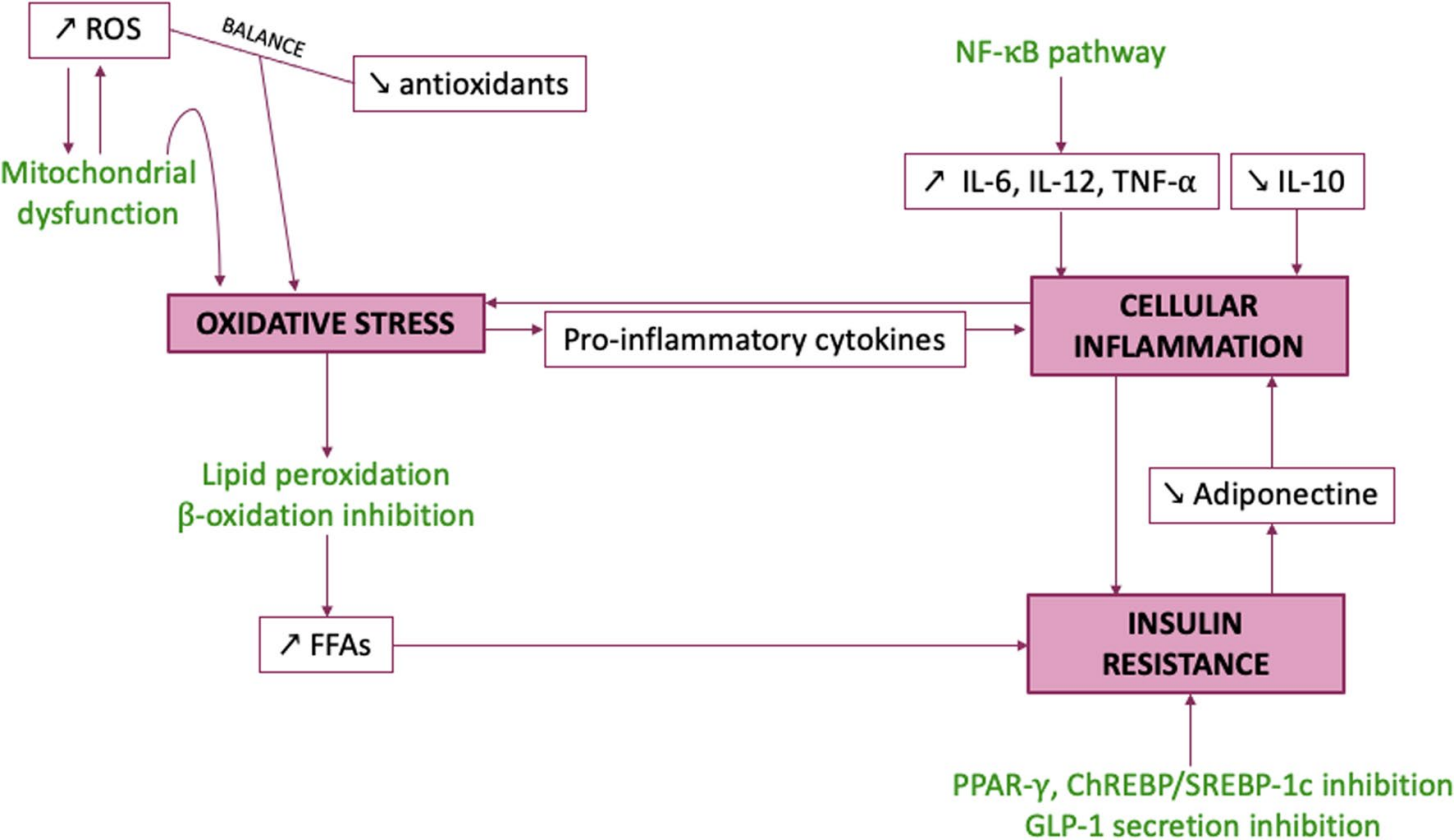
Key targets in the development and progression of NAFLD. *ChREBP* carbohydrate responsive element binding protein, *FFAs* free fatty acids, *GLP-1* glucagon-like peptide 1, *IL* interleukin, *NF-κB* nuclear factor κB, *PPAR-γ* peroxisome proliferator-activated receptor γ, *ROS* reactive oxygen species, *SREBP-1c* sterol regulatory element binding protein, *TNF-α* tumor necrosis factor

Within the complex pathophysiology of NAFLD, 3 interrelated targets appear to play determining roles in the development of the disease and its progression to NASH, oxidative stress, cellular inflammation, and insulin resistance (Fig. 3).

Oxidative stress represents a crucial process in the development of NAFLD [20], notably through induction of liver mitochondria dysfunction, which promotes lipid peroxidation and inhibits fatty acid β-oxidation [20] thereby participating in liver TGs accumulation and IR development. In addition, excessive amounts of reactive oxygen species (ROS) produced by mitochondria in NAFLD will reduce antioxidant defense through depletion of serum reduced glutathione (GSH) and mobilization of catalase (CAT) and superoxide dismutase (SOD) [40]. Mitochondrial dysfunction, depletion of antioxidants and inflammation therefore induce ROS homeostasis imbalance, which promotes worsening of hepatocyte injury, progression from NAFL to NASH, cell death and fibrosis [59].

Another key event in the progression of NAFLD is cellular inflammation. The development of adipocytes leads to increased infiltration of hepatocytes by M1-polarized macrophages secreting proteins such as IL-6, IL-12 or TNF-α in the liver, which leads to a local chronic inflammatory phase [30, 40]. This inflammation also promotes the development of IR in adipose tissue, which leads to a reduction in the serum levels of adiponectin, an adipokine that increases insulin sensitivity and decreases inflammation [40]. Inflammation can be induced by ROS via the release of proinflammatory cytokines, and conversely, inflammation is conducive to the development of oxidative stress [30]. Inflammation is mainly reduced by inhibiting the classical nuclear factor κB (NF-κB) pathway [20], which is activated by pro-inflammatory molecules such as cytokines and by oxidative stress.

A further major target would be insulin resistance, which manifests itself in adipose tissue, liver, and skeletal muscle. The ectopic accumulation of fat disturbs the insulin signal by reducing its signaling impact, and a downward circle is established since steatosis can itself promote IR within these various organs. In addition, IR can also be induced by excessive ROS production in the liver [60]. IR of adipose tissue induces the release of FFAs taken up by the pancreas into the bloodstream, resulting in a β-cell dysfunction that stimulates insulin secretion. A paradox can then be put forward; even if sensitivity to insulin decreases, its role on carbohydrates is not compromised. Insulin remains therefore able to initiate de novo synthesis of fatty acids, particularly from fructose, and promote their storage as TGs in the liver. But (i) liver IR stimulates the biochemical pathways of glycogenolysis and gluconeogenesis to increase blood glucose levels; and (ii) muscle IR reduces resorption of blood glucose, which promotes glucotoxicity, hyperglycemia being associated with diabetes development [60]. A reduction of IR can be achieved through 3 different main mechanisms: (i) via an action on the PPAR-γ receptors; (ii) via an action on SREBP-1c and on ChREBP; and/or (iii) by stimulating the release of the GLP-1 hormone via an action on the TGR5 receptors.

### Natural compounds interacting with one or more targets and evidence supporting their use in the management of NAFLD

**Fig. 4:**
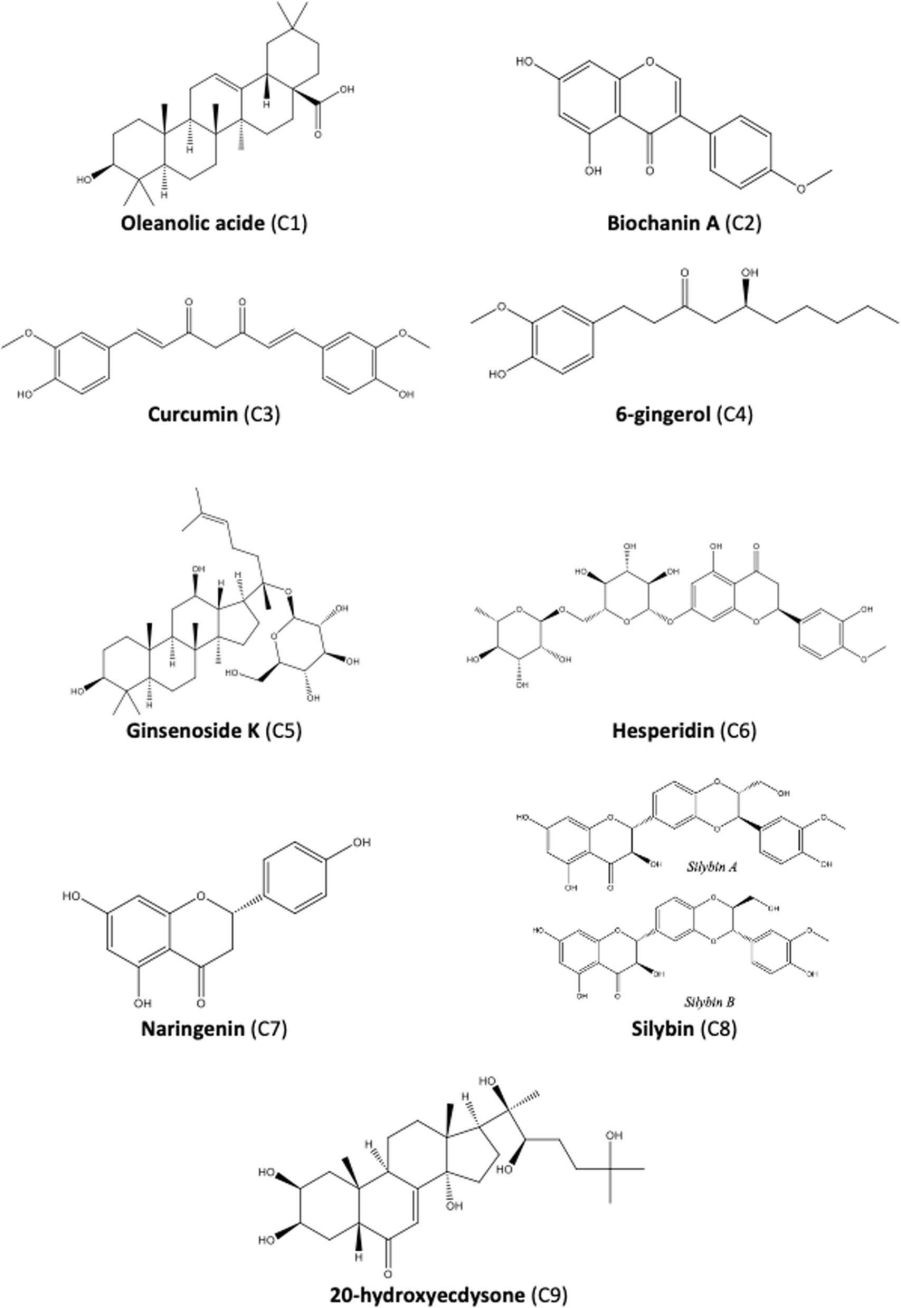
Chemical structures of natural compounds. *C* compound

In this section, we will present the clinical, in vivo, and in vitro evidence for various natural compounds influencing NAFLD. Table 3 provides a concise summary of the key effects of the compounds presented in this study, including the supporting evidence levels and the pharmacological targets they impact. The studies highlighted elucidate the advantageous impacts of several natural compounds on the three previously discussed targets: oxidative stress, cellular inflammation, and insulin resistance (IR). The molecular structures of these compounds are depicted in Fig. 4.

**Table 3:** Summary of results for the different naturally occurring molecules used

Naturally occurring compounds	Plants	Levels of evidence/models studied	Pharma-cological targets	Doses	Main results	References
Oleanolic acid	*Olea europaea* L.	In vitro: STC-1 murine enteroendocrine cell lines	TGR5	Oleanolic acid at 10 μM	Increase in GLP-1 secretion by (4.1 ± 0.6) times over basal levels	[61]
		In vitro: STC-1 murine enteroendocrine cell lines transfected with TGR5 sRNAi			Inhibition of GLP-1 secretion by (78 ± 10) % compared to transfected cells with a control sRNAi	
		In vivo: rats with diet induced metabolic syndrome and NAFLD	NF-κB SREBP-1c	25, 50 or 100 mg/kg for 8 weeks	Inhibition NF-κB of and suppression of SREBP-1c expression	[62]
Biochanin A	*Origanum vulgare* L. *Trifolium pratense* L.	In vivo: streptozotocin-induced diabetic rats after a young	PPAR-γ	10 mg/kg/day of biochanin A	Reduction in blood glucose levels by 49.72% after 45 days	[70]
		In vivo: streptozotocin-induced diabetic rats after a fat-enriched diet		10, 20 and 40 mg/kg/day of biochanin A	Dose-dependent decrease in blood glucose levels	[41]
Curcumin	*Curcuma longa* L.	Clinical trial: double blind RCT on 50 patients with NAFLD $\ge$ 18 years old	NF-κB TNF-α	3 capsules at 500 mg/day of BCM95 for 12 weeks	Inhibition of NF-κB and statistically significant decrease in serum TNF-α levels but no difference compared to control group (lifestyle modification)	[74]
		Clinical trial: double blind RCT on 50 patients with NAFLD $\ge$ 18 years old	PPAR-γ	3 capsules at 500 mg/day of BCM95 for 12 weeks	Statistically significant decrease in serum AST and blood glucose levels but no difference compared to the control group (lifestyle modification)	[75]
6-Gingerol	*Zingiber officinale* Roscoe	In vivo: rats with IR fed with a high-fat and a high-carbohydrate diet	PPAR-γ SREBP-1c ChREBP	100 or 200 mg/kg of total ginger extract for 10 weeks	Decrease in blood glucose levels and increase in insulin concentration to 200 mg/kg	[86]
		In vitro: human hepatoma cells HepG2	TNF-α IL-6	25, 50 and 100 mg/kg of 6-gingerol for 8 weeks	Decrease serum TNF-α and IL-6 levels at 100 mg/kg	[83]
		In vivo*:* hamsters fed with a high-fat diet	IκB NF-κB	100 mg/kg of 6-gingerol for 8 weeks	Dose-dependent reduction of IκB degradation, the repressor of NF-κB	
		In vivo: streptozotocin-induced diabetic rats	CAT GPX SOD	0.5, 1 and 5% of ginger powder in rat feed for 4 weeks	Increase of CAT, GPX and SOD activity at a dose of 5%	[88]
		Clinical trial: double blind RCT on 44 patients with NAFLD	–	2 g/day of ginger supplement for 12 weeks	Decrease in serum ASAT and cytokine levels, decrease of ISI and NAFLD grade compared to control group	[89]
Ginsenoside K	*Panax ginseng* C.A. Meyer	In vitro: NCI-H716 human enteroendocrine cell lines	TGR5	Ginsenoside K at 10, 50 and 100 μM	Increase in GLP-1 secretion at 100 μM	[98]
		Clinical trial: double blind RCT on 400 prediabetic and obese patients $\ge$ 18 years old	–	2 capsules of GINST15 at 160 mg hydrolyzed ginseng (including ≥ 5 mg/g ginsenoside K) for 12 months	Reduction in fasting blood glucose levels and increase in postprandial insulin concentration compared to the control group	[97]
Hesperidin	*Citrus aurantium* L.	In vivo: Wistar albino male rats	CAT GPX SOD GST GSH	20, 40 and 80 mg/kg of hesperidin for 10 days	Increase of CAT, GPX, SOD and GST activity and increase of GSH levels	[100]
		Clinical trial: double blind RCT on 50 patients NAFLD between 18 and 70 years old	NF-κB TNF-α	2 capsules of hesperidin at 500 mg for 12 weeks	Reduction in inflammatory markers and blood glucose levels compared to the control group	[105]
Naringenin	*Citrus aurantium* L.	In vivo: rats with arsenic-induced hepatic lesions	CAT GPX SOD GSH	50 mg/kg of naringenin for 2 weeks	Increase of CAT, GPX and SOD activity, and increase of GSH levels	[101]
		In vivo: Wistar albino rats with ethanol-induced liver damage	NF-κB TNF-α IL-6 COX2	50 mg/kg of naringenin for 60 days	Inhibition of NF-κB and decrease in serum TNF-α, IL-6 and COX2 levels	[108]
Silymarin	*Silybum marianum* L. Gaertner	In vivo: rats with arsenic-induced hepatic lesions	CAT GPX SOD GSH	50 mg/kg of silymarin for 2 weeks	Increase of CAT, GPX and SOD activity, and increase of GSH levels	[101]
		In vitro: NCI-H292 human cell lines	NF-κB TNF-α	1, 10 and 100 μM	Inhibition of NF-κB and decrease of TNF-α levels	[117]
		Meta-analysis of 8 RCT: patients with NAFLD > 18 years old	–	70–540 mg/day of silymarin for 8–24 weeks depending on the RCT	Decrease in serum ASAT levels of 6.57%	[123]
Quinoa	*Chenopodium quinoa* Willd.	In vitro: RAW 264.7 murine macrophage cells	TNF-α IL-6	50, 100 or 200 μg/mL of quinoa saponins	Decrease TNF-α and IL-6 levels	[129]
		In vivo: diet-induced obese female mice	GSH SREBP-1c	2 g/day of quinoa for 12 weeks	Increase of GSH levels and lowers transcription of SREBP-1c	[130]
		Clinical trial: prospective double blind random study on 35 overweighted menopaused women	–	25 g/day of quinoa flakes for 4 weeks	Decrease in triglycerides and cholesterol levels, potential protection against oxidative stress	[133]
		Clinical trial: double blind RCT on 50 overweight patients	–	25 and 50 g/day of quinoa for 12 weeks	Reduction of triglycerides blood content	[134]

*ASAT* aspartate aminotransferase, *CAT* catalase, *ChREBP* carbohydrate responsive element binding protein, *COX2* cyclooxygenase 2, *GLP-1* glucagon-like peptide 1, *GPX* glutathione peroxidase, *GSH* reduced form glutathione, *GST* glutathione-S-transferase, *IL-6* interleukin 6, *IR* insulin resistance, *ISI* insulin sensitivity index, *NAFLD* non-alcoholic fatty liver disease, *NF-κB* nuclear factor κB, *PPAR-γ* peroxisome proliferator-activated receptor γ, *RCT* randomized controlled trial, *sRNAi* small interfering RNA, *SOD* superoxide dismutase, *SREBP-1c* sterol regulatory element binding protein, *TGR5* G protein-coupled receptor 5, *TNF-α* tumor necrosis factor

In murine enteroendocrine cells, oleanolic acid (**C1**), a pentacyclic triterpenoid widely distributed in plants, significantly stimulates GLP-1 secretion via activation of the TGR5 receptor [61]. Suppression of TGR5 expression by small interfering ribonucleic acids (sRNAi) inhibited GLP-1 secretion, providing evidence that oleanolic acid-induced GLP-1 secretion is mediated by the TGR5 receptor [61]. On rats with diet-induced metabolic syndrome and NAFLD, oleanolic acid (i) enhances insulin sensitivity; (ii) improves glucose tolerance; (iii) triggers a reduction in NF-κB activation, by limiting p65 phosphorylation, which attenuates the NF-κB inflammatory pathway and prevents the development of NASH; (iv) acts as a regulator of liver lipid metabolism protein expression; (v) suppresses the expression of SREBP-1c, FAS, ACCα and LXRα, which are involved in hepatic lipid metabolism; (vi) suppresses the transcription of lipid transport proteins such as FABP1, FABP4 and CD36; and (vii) improves the gut microbiota by reducing endotoxemia and protecting the intestinal barrier, leading to an enrichment of the pool of butyrate-producing bacteria [62].

Biochanin A (**C2**), an *O*-methylated isoflavone belonging to the phytochemical class of flavonoids, has several pharmacological effects, including anti-inflammatory, lipid-lowering, and cholesterol-lowering effects as well as cancer chemoprevention-related effects [63, 64]. In diabetic rats, biochanin A, as a potent activator of both PPAR-γ and PPAR-α receptors, has shown promising and interesting effects in lowering glycemia and improving insulin sensitivity [65, 66]. Biochanin A binds to the PPAR-γ receptor and induces transcription of target genes via selective interactions between cofactors and the receptor [67, 68]. As a partial agonist, biochanin A may have fewer side effects than the full agonists thiazolidinediones [68–71].

Curcumin (**C3**) is a polyphenol that possesses several biological activities of interest in the context of NASH, notably by reducing inflammation, modifying lipid profiles, and increasing insulin sensitivity. These properties are notably due to interactions between curcumin and transcription factors (NF-κB), enzymes (COX2), receptors (PPAR-γ) and cytokines (IL-6, TNF-α) [72]. An in vivo study on NASH models demonstrated the antioxidant properties of curcumin through upregulation of the Nrf2 gene [73]. Clinical trials on NAFLD patients confirmed the anti-inflammatory [74] and antidiabetic [75] effects of curcumin via NF-κB inhibition, a significant decrease in serum TNF-α, ASAT and glucose levels as well as an increase in insulin levels. By contrast however, recent clinical studies indicate that curcumin combined with lifestyle modification would have no superior effect compared to lifestyle modification alone. The very low bioavailability of curcumin may explain this [76]. Its bioavailability can however be enhanced through galenics, e.g. in micronized micelles [77], or by association with the alkaloid piperine [78] and it may be appropriate to evaluate these high-availability dosage forms. However, special care should be taken with these modified forms of curcumin, as several cases of hepatitis have been reported [79].

6-Gingerol (**C4**), an alkylated phenol from ginger, has various pharmacological effects, including a reduction of inflammatory factors and oxidative stress, an improvement of lipid profile and a reduction of glycemia [80, 81]. Several recent studies have shown that the lipid-lowering and antidiabetic effects of 6-gingerol are mediated by PPAR receptors [82, 83], notably the PPAR-γ receptor, allowing it to induce hypoglycemic and anti-hyperglycemic effects via a decrease in the expression of SREBP-1c and ChREBP [84, 85]. An in vivo study on rats with high-fat, high-carbohydrate diet-induced IR showed a significant reduction in blood glucose levels as well as an increase in insulin sensitivity after administration of a high dose of total ginger extract (200 mg/kg; [15.6 ± 0.5]% of 6-gingerol) [86]. Furthermore, anti-inflammatory effects of 6-gingerol are mediated by inhibition of the classical NF-κB pathway [87]. In an in vitro study on hepatoma HepG2 cells, 6-gingerol dose-dependently induced a significant reduction in TNF-α and IL-6 levels and, in high-fat diet hamsters, it reduced the degradation of IκB, the repressor of NF-κB [83]. In streptozotocin-induced diabetic rats, the administration of ginger powder decreased glycemia, improved TG profile in a dose-dependent manner and significantly increased CAT, GPX and SOD activities [88]. All these effects have been confirmed by a randomized, double-blind clinical study in patients with NAFLD. Indeed, this study showed that a ginger powder supplement decreased serum ASAT, cytokine levels, insulin sensitivity index and NAFLD grade compared to control group [89]. In addition, two in vivo assays suggested that miR-107-3p regulation by 6-gingerol reduces inflammation and lipid accumulation and enhances mitochondrial function, but further studies are needed to clarify its role [90, 91].

When ingested orally, ginsenosides, ginseng saponosides, are transformed into ginsenoside K (**C5**) by the gut microflora, the main active metabolite absorbed into the bloodstream. In rats, both in vitro and in vivo, hepatoprotective, anti-inflammatory and antifibrotic effects of ginsenoside k were ascribed to both an activation of AMPK and a repression of mTOR [92–96]. Additionally, ginsenoside K exhibits antidiabetic effects by inhibiting apoptosis of pancreatic islet β-cells and by stimulating insulin secretion [97, 98]. In human enteroendocrine cell lines, ginsenoside K led to a significant increase in GLP-1 secretion through activation of the TGR5 receptor, with a highest effect at 100 μM [98]. In addition, a randomized clinical trial showed that treatment of prediabetic patients with fermented ginseng (i.e. ex vivo metabolization of part of the natural ginsenosides mix to ginsenoside K) induced a reduction in fasting blood glucose and an increase in postprandial insulin concentration [97]. It seems that fermented ginseng allows faster and higher absorption of ginsenoside K and is well tolerated, except for some cases of mild diarrhea [97].

The flavanones hesperidin (**C6**) and naringenin (**C7**) from *Citrus aurantium* L. are among the most potent hepatoprotective flavonoids that certainly contribute to the pharmacological effects of *Citrus aurantium* in the treatment of liver disease [99]. Their antioxidant properties are mediated by an increase in GSH levels and in CAT, GPX, SOD, and GST activities [100]; Naringenin also exerts its antioxidant effects via an increase in the expression of nrf2 [101]. Hesperidin could also exert its anti-inflammatory properties through a decrease in cytokine levels, inhibition of the NF-κB pathway and inhibition of the endoplasmic reticulum stress (ERS)-induced inflammatory pathway [102]. Several studies have hypothesized that hesperidin improves steatosis in vitro and in vivo through AMPK activation [103, 104]. Interestingly, short-term randomized clinical trials on NAFLD patients demonstrated a significant decrease in inflammatory factors and glycemia in the hesperidin-treated groups [105, 106]. These advantages should however be balanced with reports associating this compound with adverse cardiovascular disorders [99, 107].

The anti-inflammatory effects of naringenin are manifested via a decrease in the production of pro-inflammatory cytokines (TNF-α and IL-6 in particular), an inhibition of the classical NF-κB pathway and a decrease in COX2 expression [108]. A randomized clinical trial investigated the effects of daily 200 mg naringenin treatment on obese NAFLD patients and demonstrated an improvement in their lipid profiles [109].

The methanolic extract of Milk Thistle (*Sylibum marianum* L. Gaertner), called silymarin, is a mixture of at least 8 flavolignans [110], from which Silybins A and B are among the major compounds (**C8**) [111]. The oral bioavailability of silymarin flavolignans is relatively low, mainly due to their poor intestinal resorption, low aqueous solubility, high hepatic first-pass effect and rapid excretion in bile and urine [112]. Fortunately, several formulation strategies have been studied to increase the bioavailability of sylimarin [113], including phytosome based on the formation of a complex with phosphatidylcholine [114].

Silybins exert metabolic, antioxidant and anti-inflammatory effects, the 3 major targets considered important in NAFLD. Indeed, in vivo studies on NAFLD models lead to a significant improvement in inflammation and NAFL through (i) a decrease in serum lipid levels, due to stimulation of FFAs β-oxidation and action on the PPAR-α receptor; (ii) an improvement in insulin sensitivity following activation of the PPAR-γ receptor; (iii) an increase in oxidative stress via stimulation of endogenous antioxidants (GSH, CAT, GPX and, SOD); and (iv) a decrease in inflammation through inhibition of NF-κB and reduction in TNF-α levels [115–122]. Another study conducted in rats with arsenic-induced liver damage demonstrated the effect of silymarin on antioxidant enzymes and its antihepatotoxic activity [101]. A meta-analysis of 8 randomized control trials in NAFLD patients [123] indicates a decrease in serum aspartate transaminase (ASAT) levels, considered as a restoration of liver function. The dosage regimen showing the most significant effect was 280 mg/day of silymarin for 24 weeks [123]. This meta-analysis also showed that silymarin is well tolerated and could therefore be of great interest in the treatment of NAFLD, especially NASH. Several recent clinical trials have also supported these findings [124, 125].

*Chenopodium quinoa* Willd. is a pseudocereal recognized for its high nutritional value. The plant methanolic extract contains a significant content of bioactive phytochemicals such as terpenoids, betanins, carotenoids, polyphenols, saponins, and phytoecdysteroids [126]. Several studies have recently described quinoa as a highly promising functional food component to treat obesity and metabolic diseases such as metabolic syndrome, T2DM and NAFLD [125–127]. A study investigating the effects of quinoa intake on high-fed rats suggests that quinoa phytochemicals are the causes of lipid metabolism dysfunction regulation, leading to a resolution of IR, liver steatosis decrease, and anti-inflammation and antioxidative quinoa properties [128]. An in vitro study reported that quinoa saponins inhibit inflammatory properties by preventing the release of TNF-α, IL-6 and NO in lipopolysaccharide-induced RAW264.7 cells [129]. An in vivo study on diet-induced obese female mice suggested that quinoa increases GSH levels and lowers transcription factors regulating lipogenesis (PPAR-γ, SREBP-1c, AP2, cEBP-α, -β et-γ) [130]. Flavonoids and phenolic acids, belonging to the polyphenol family, present antioxidant, anti-inflammatory and anti-obesity properties [131, 132]. Moreover, findings from two double-blind clinical trials with overweight individuals demonstrated that quinoa intake decreased levels of triglycerides (TG) and cholesterol, suggesting a protective effect against oxidative stress [133, 134]. 20-Hydroxyecdysone (20-E) (**C9**) is the most abundant phytoecdysteroid found in *Chenopodium quinoa* and has demonstrated some antidiabetic properties in high-fed rats [135]. A study demonstrated that ethanol quinoa leachate, containing 60% of the total amount of 20-E of untreated quinoa seeds, lessens blood glucose levels in diet-induced obese, hyperglycemic mice [136]. 20-E could also be the major factor responsible for weight lowering and lipid profile improvement effects of quinoa [137, 138]. 20-Hydroxyecdysone is found in edible crops such as spinach (*Spinacia oleracea* L.) and in the highest concentration in the seeds of *Chenopodium* quinoa Willd. [136]. To elaborate a treatment of NAFLD based on this highly interesting plant, more studies are needed to deepen the understanding of quinoa effect mechanisms.

Most of the current clinical studies presented in this paper have been carried out on a small number of patients. Additionally, whether these molecules will indeed reach the desired target in vivo and whether the active plasmatic and tissular concentration will be reached after resorption remain major questions. The clinical relevance of some effects then remains quite uncertain, especially when the dose/concentration-effect relationships have not really been studied. Thus, the aim of this review is not to recommend the use of these natural compounds, as more evidence is still needed, but to give readers an overview of the clinical studies that have already been carried out. The use of these plants in the treatment of NAFLD could be suggested after conducting clinical trials that robustly affirm their efficacy and safety.

## Conclusion

Given the high prevalence of NAFLD and the serious complications that this disease can cause, it is important to encourage patients to be diagnosed but also to assist them in managing the disease, notably through hygieno-dietetic measures. For both prevention and treatment, lifestyle modifications involving diet and exercise undoubtedly constitute the first line treatment. Nonetheless, patients’ adherence to these recommendations is generally poor. As there are no drugs with a marketing authorization for this indication yet, naturally occurring compounds, given their safety and activity profiles, appear as an attractive alternative for treating NAFLD. The present review therefore focused on the identification of natural compounds and extracts that may play a role in this disease. The common use in traditional medicine of the plants from which these molecules are derived, the absence of serious adverse effects of most of them and the available in vitro, in vivo*,* and clinical data reported so far make those compounds interesting prospects for the management of NAFLD.

But, even if most of these herbal compounds are already marketed in pharmacies, they are available in many different forms and presentations (plant powder, various extracts) for which the phytochemical profiles can markedly differ, making their effects uncertain. Further studies are needed to investigate their likely benefits in patients with NAFLD in order to develop an effective herbal armamentarium.

## Data Availability

Data sharing not applicable to this article as no datasets were generated or analysed during the current study.

## Abbreviations

AFLD: Alcoholic fatty liver disease
BMI: Body mass index
HCC: Hepatocellular carcinoma
ELF test: Enhanced liver fibrosis test
FIB-4 index: Fibrosis-4 index
FLI: Fatty liver index
^1^H-MRS: Magnetic resonance proton spectroscopy
MAFLD: Metabolic disfunction-associated fatty liver disease
MBOAT7-TMC4: Membrane-bound *O*-acetyltransferase domain containing 7-trans-membrane channel-like 4
MRE: Magnetic resonance elastography
MRI: Magnetic resonance imaging
NAFL: Non-alcoholic fatty liver
NAFLD: Non-alcoholic fatty liver disease
NASH: Non-alcoholic steatohepatitis
NFS: NAFLD fibrosis score
PNPLA-3: Patatin-like phospholipase domain-containing protein 3
TLR4: Toll like receptor 4
